# UV-Initiated Crosslinking Reaction Mechanism and Electrical Breakdown Performance of Crosslinked Polyethylene

**DOI:** 10.3390/polym12020420

**Published:** 2020-02-12

**Authors:** Yu-Wei Fu, Wei-Feng Sun, Xuan Wang

**Affiliations:** Key Laboratory of Engineering Dielectrics and Its Application, Ministry of Education, School of Electrical and Electronic Engineering, Harbin University of Science and Technology, Harbin 150080, China; yuwei_fu@126.com

**Keywords:** crosslinked polyethylene, photon initiator, crosslinking reaction, first-principles calculation, dielectric breakdown field

## Abstract

The ultraviolet (UV) irradiation crosslinking reactions of polyethylene and the electronic properties of photo-initiators and reaction products are theoretically investigated by the first-principles calculations. The crosslinked polyethylene (XLPE) materials are prepared in experiments that employ the UV-initiated crosslinking technique with different photon-initiation systems. Infrared spectrum and the alternating current dielectric breakdown strength of UV-initiated XLPE are tested to explore the effect of reaction products on the breakdown characteristics in combination with the electron structure calculations. The theoretical calculations indicate that the 4-hydroxybenzophenone laurate, which is compatible with polyethylene, can effectively initiate crosslinking reactions of polyethylene molecules under UV photon excitation and will produce reaction by-products from carbonyl radicals; as a macromolecular auxiliary crosslinker, the monomer or homopolymer of dioleyl-2,2′,4,4′-tetraallyl isocyanurate can form chemical connections with multiple polyethylene molecules acting as a crosslinking node in a photon-initiated reaction process. The carbonyl, hydroxyl, or ester groups of reaction by-products are capable of capturing hot electrons to prevent polyethylene molecules from impact ionization, and thus will increase the breakdown electric field. The macromolecular auxiliary crosslinker and the macromolecular photon initiator as well as its reaction by-product can convert the energy of their captured high-energy electrons into heat, which can act as a voltage stabilizer. The molecule characterization of infrared spectra demonstrates that the characteristic absorption peaks of the carbonyl in the macromolecular photon initiator and the allyl in the macromolecular auxiliary crosslinking agent are gradually decreasing in intensity as the crosslinking reaction proceeds, which is consistent with the conclusion from theoretical calculations. Compared with the small molecular photon-initiation system generally used in the photon-initiated crosslinking process, the higher dielectric breakdown field of XLPE being prepared by utilizing a macromolecular photon-initiation system is in good agreement with the calculation results of electronic affinity and ionization potential. The consistent results of the experiments and first-principles calculations elucidate the fundamental mechanism of the UV-initiation crosslinking technique and suggest a prospective routine to improve the insulation strength for developing high-voltage XLPE insulating materials.

## 1. Introduction

At present, submarine power cables are primarily fabricated by using crosslinked polyethylene (XLPE) materials to realize electrical insulation in high voltage grade [1,2]. However, the traditional crosslinking technology using peroxide in the production of high voltage cables has shown a variety of inevitable limitations in the heating processes of chemical reactions, such as high energy consumption, low heat transfer efficiency, and short-time continuous reaction. In particular, the scorching phenomenon frequently occurs at the blind corner of mold in the traditional production process using the chemical crosslinking method, which will cause substantial defects in the insulation layer of the cable and thus deteriorate the electrical performance under high-voltage. Therefore, the continuous cable production employing the chemical crosslinking technique with peroxide can not persist for sufficiently long time, which limits its manufacture to obtain a single cable with enough length for avoiding redundant connections of relaying cables. For submarine cables, the length of a single cable is expected to be increased as much as possible to reduce the number of intermediate joints, which is the most vulnerable area to failure, thereby improving the operation reliability and abating the maintenance cost of submarine cables. The ultraviolet (UV) crosslinking technique is a non-heat-sensitive process of fabricating XLPE with the advantages of a high production rate, long-time continuous production, small fundamental investment, low raw material cost, and low energy consumption, which is promised to realize the industrial production of high voltage grade and extra-long XLPE insulated cables [3,4,5,6]. The photon-initiation systems used for the UV polyethylene crosslinking reaction normally consist of small molecules with high photon-initiation efficiency, which however show inevitable disadvantages in practical applications. The poor compatibility with polyethylene (PE) of the small photon initiator and auxiliary crosslinker molecules makes them more apt to migrate out to the surface of the polymeric blend at room temperature, which will decrease the reaction rate and crosslinked degree of XLPE. Furthermore, small molecular initiation systems with high volatility are prone to be evaporated and deposit on the UV lamp cover in the crosslinking process, which will decrease the UV light transmission efficiency and corrode the irradiation equipment [7,8,9]. Therefore, it is urgent to develop a novel photon initiator and auxiliary crosslinking agent with low volatility, preferable compatibility with PE, and high photon-initiation efficiency. The volatility of the photon-initiation system can be considerably reduced by introducing long-chain alkyl groups into the small molecules [10,11]. Some additives with the C_8_–C_16_ alkyl groups represent high compatibility with the PE matrix, which can inhibit the thermal diffusion of the photon initiator and crosslinking agent in PE [12].

The previous studies on the mechanisms of photon-initiated crosslinking reactions are mainly based on experiments. Chen investigated the initiating functions of different photon initiators and suggested that benzophenone (BP) and its derivatives can present high photon-initiation efficiency [13]. It has been reported that the majority of PE crosslinking reactions occurs on secondary and tertiary carbon atoms [14,15]. The auxiliary crosslinking mechanism of triallyl cyanoureate (TAC) was elucidated as that the auxiliary crosslinking agents with allyl group can self-polymerize to homopolymer with a small molecular weight at high temperatures [16,17,18]. The crosslinking structure of the polymer is related to the double-bond conversion rate and crosslinking degree [19]. The allyl groups in the auxiliary crosslinkers between PE macromolecules will produce a crosslinked structure in a bridged or star configuration [20]. Nevertheless, it is difficult to observe in experiments how the active free radical intermediates are produced in the photon-initiated crosslinking reactions of polyethylene. With the development of quantum mechanics and computer science, computational chemistry has gradually become an important method to guide scientific research in the process of exploring the chemical reaction mechanism. Quantum chemistry calculations of the PE crosslinking reaction initiated by BP under UV irradiation have shown that the produced XLPE comprises the carbonyl, phenyl, alkoxy, and heteroatomic groups [21,22]. The crosslinking mechanism of the auxiliary crosslinking agent has been revealed that Triallyl isocyanurate (TAIC) could accelerate the accomplishment of crosslinking reactions from minutes to seconds [23]. The quantum mechanics calculations can lay a theoretical foundation for exploring the mechanism and application of the photon-initiated crosslinking reaction.

In the present study, the excited state energy of the 4-hydroxybenzophenone laurate (BPL) is calculated firstly, and the transition state, energy barrier, and Gibbs free energy of various chemical reactions are then calculated according to the UV irradiation crosslinking principle, by which the crosslinking reaction mechanism of dioleyl-2,2-,4-,4-tetraallyl isocyanurate (STAIC) as an auxiliary crosslinking agent is particularly analyzed with the consideration of by-product effects on insulation performance. The effects of using different photon-initiation systems on the insulation performance of XLPE are eventually analyzed by calculating the electronic structures of photon initiators, auxiliary crosslinking agents, and by-products of photon-initiated crosslinking reactions. In order to verify the theoretical calculation results, the corresponding XLPE materials are prepared with the UV-initiated crosslinking technique, and the infrared spectrum tests for characterizing molecular structures and the electrical breakdown experiments are performed to elucidate the reaction mechanism of the crosslinking reaction with a macromolecular photon-initiation system. The type and content of the photon-initiation system will have a considerable impact on the crosslinked structure, mechanical, and electrical properties of XLPE materials, especially for the reactants that do not contribute to the crosslinked network structure [24,25]. Therefore, the crosslinking mechanism of the macromolecular photon-initiation system and its effect on the dielectric breakdown field are studied in this paper, which can be referenced to optimize the UV-initiated crosslinking process of XLPE and improve the electrical properties of XLPE by reasonably designing the molecular structure of the macromolecular photon initiator and crosslinking agent for promoting the PE crosslinking efficiency of photon-initiation technique.

## 2. Theoretical Methodology and Experiments

### 2.1. Polyethylene Photon-Crosslinking Reaction Mechanism

**Figure 1:**
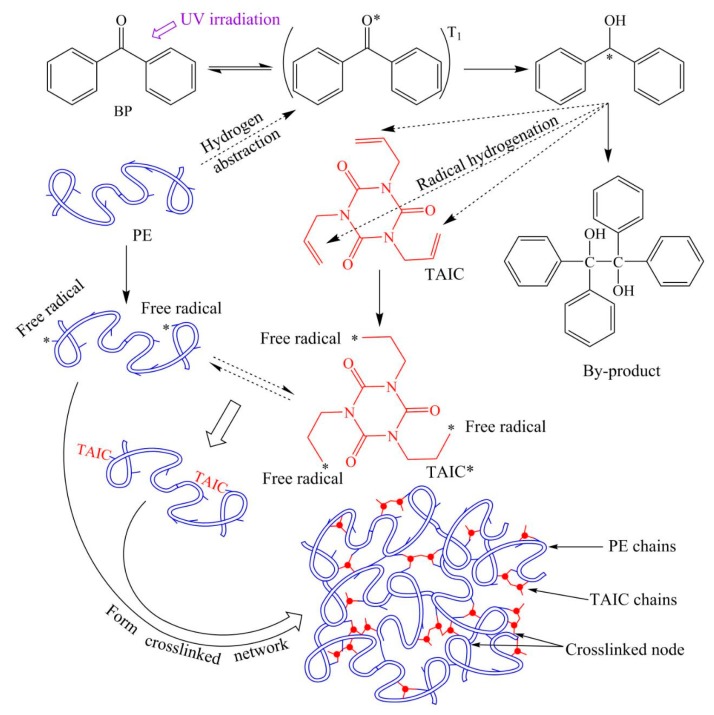
Schematic crosslinking reaction of polyethylene initiated by UV irradiation.

The UV-initiated crosslinking process of polyethylene molecules is fulfilled, taking advantage of the UV transparency of low density polyethylene (LDPE) fluid at the temperature higher than melting point in actual experiments and UV-XLPE productions, as that the UV lights are directly incident through the melting mixture of the LDPE, photon initiator, and auxiliary crosslinking agents. Figure 1 schematically illustrates the representative UV-initiated crosslinking reactions of polyethylene molecules by using photon initiator BP and auxiliary crosslinking agent TAIC. Firstly, the photon initiator (PI) is instantaneously excited to the first triplet (*T*_1_) state which has just transited from the single states acquired by UV irradiation, and subsequently initiates the hydrogen abstractions from PE molecules to the excited PI in *T*_1_ state, resulting in the chemical radicals on PE molecules and the hydrogenated radical PI molecules. Then, the partial fractures of carbon double bonds on auxiliary crosslinking agent (AC) molecules are almost simultaneously triggered by the donation of hydroxy hydrogen from the hydrogenated radical PI molecules. Eventually, the considerable amounts of transient free radicals on the molecules of both PE and AC bond chemically to each other with the statistic probabilities determined by reaction kinetics to accomplish crosslinking reaction in three PE-crosslinking configurations of PE-PE, PE-AC-PE, and PE-[AC]-PE ([AC] represents the copolymer segment of AC) [26,27]. Macroscopically, the various free radical chains of polymer molecules will react to finally form a crosslinked network, as shown in the bottom of Figure 1. It should be noted that a small number of PI molecules will further form reaction by-products after hydrogen abstraction from polyethylene, as shown at top right part of Figure 1. Without an auxiliary crosslinking agent, the polyethylene macromolecules can only form the crosslinking nodes through the chemical bonding by their own free radicals. In contrast, the auxiliary crosslinking agent in the form of monomers or homopolymers represents higher ability in the participation of forming polyethylene crosslinking nodes, which can increase the crosslinking probability and thus improve the photon-initiated crosslinking efficiency [28].

### 2.2. Molecular Model and Calculation Schemes

A molecular model in randomly distributed torsion of the symptomatic PE molecule polymerizing in 30 degree with the equilibrium C–C and C–H bond lengths of 1.50 Å and 1.10 Å respectively is initially constructed by the rotational isomeric state (RIS) method. The hydrogen abstraction reactions are supposed to occur at the middle position of PE backbone chain. According to the all-electron numerical-orbit first-principles scheme based on density functional theory (DFT), the DMol3 code of Materials Studio 8.0 software package is used to calculate the UV-initiated crosslinking reactions and the electronic structures of reacting molecules [29,30]. The relaxed molecular structures are calculated by minimizing the total energy with a conjugated gradient algorithm [31] in the geometric optimization of reactants, transition states, and products, in which the total energy convergence of 1.0 × 10^−5^ Ha/atom is required with an atomic force and displacement less than 0.002 Ha/Å and 0.005 Å, respectively. The exchange–correlation interaction of electrons is described by the M11-L energy functional of meta-generalized gradient approximation (meta-GGA) [32]. The wave functions of electron eigenstates are expanded by a double numerical polarized (DNP) basis set with the global orbital cutoff being set as 5.0 Å to adequately decrease the error caused by the finite basis set. The all-electron-relativistic core treatment is utilized to evaluate the interaction between electrons and atomic core. The direct inversion in an iterative subspace (DIIS) cheme of density mixing is adopted to speed up electron relaxations in self-consistent field (SCF) iterations with the charge mixing amplitude and SCF tolerance being set as 0.2 and 1.0 × 10^−6^ Ha/atom (1 Ha = 27.2 eV), respectively [33]. The linear/quadratic synchronous transit (LST/QST) algorithm is employed to search the transition states in chemical reactions, in which the reaction system is firstly minimized in energy by LST, followed by repeated conjugate gradient minimization and QST maximization until the transition state is determined. The transition state is confirmed by normal modes of atomic vibrations, which will represent one imaginary frequency corresponding to the stretching vibration of coupling the broken and forming bonds. Reactants and products are also verified by the real frequency of harmonic oscillation modes. The climbing-image nudged elastic band (CI-NEB) method is used to calculate the minimum energy path (MEP) of valid processes in chemical reactions [34]. The singlet and triplet excitation states of photon initiators are calculated by the time-dependent DFT (TD-DFT) scheme using the adiabatic local density approximation (ALDA) kernel with exchange–correlation terms being included.

The vibrational analysis calculations in geometry optimization are used to compute the important thermodynamic properties such as enthalpy (*H*), entropy (*S*), and free energy (*G*). The internal energy difference (Δ*U*) of the system arises from the electronic (Δ*E*_tot_), vibrational (Δ*E*_vib_), translational (Δ*E*_trans_), and rotational (Δ*E*_rot_) contributions, as given by the following equation:

(1) $$\Delta U=\Delta E_{\mathrm{tot}}+\Delta E_{\mathrm{vib}}+\Delta E_{\mathrm{trans}}+\Delta E_{\mathrm{rot}}$$

The Gibb’s free energy difference in chemical reactions is addressed as:

(2) $$\Delta G^{T}=\Delta H^{T}-T\Delta S^{T}$$

where Δ*H^T^* and Δ*S^T^* denote enthalpy and entropy differences, respectively, both of which can be directly derived from internal energy, as described in Equation (2). Once the ground-state structure is determined and the vibrational frequencies of normal modes are obtained using statistical mechanics, all the terms in Equations (1) and (2) can be calculated as implemented by DMol3 code.

The energy gap (*E*_g_) between the highest occupied molecular orbital (HOMO) and lowest unoccupied molecular orbital (LUMO), the ionization potential (IP), and the electron affinity (EA) are achieved from electronic structure and total energy calculations. The vertical ionization potential IP(v) is defined as the difference between the total energies of cation and neutral molecule in the geometry as neutral equilibrium structure, while the adiabatic ionization potential IP(a) is equal to the energy difference between the cation and neutral molecule both in optimized geometries with the lowest energy. The vertical electron affinity EA(v) is determined by the energy difference between a neutral molecule and anion with the identical equilibrium geometry as the neutral molecule, while the adiabatic electron affinity EA(a) is calculated both from the optimized geometries of neutral molecule and anion.

### 2.3. Material Preparation and Test Methods

Employing two combinations of photon initiator and auxiliary crosslinking agent with multiple functional groups, the XLPE materials are prepared by the UV-initiated crosslinking technique. The raw materials in PE crosslinking experiments are used as follows: LDPE (LD200GH, Sinopec Company Ltd., Beijing, China) as the matrix material, photon initiator (BP or BPL, Jinleiyuan Chemical Co., Ltd., Lianyungang, China), and auxiliary crosslinking agent (TAIC or STAIC, Nobel Company Ltd., Aksu, China). In the melting blend process of preparing the initial mixtures, the pristine LDPE materials are heated to be melted at the temperature of 120 °C with a stirring speed of 60 rpm in a Torque Rheometer (RM200C, Hapro Company Ltd., Harbin, China) for 1 min, and then 3 wt % AC and 0.2 wt % PI are added, being blended for 3 min and cooled down to room temperature. In the process of photon-initiated crosslinking reactions, the prepared mixtures are firstly melted in a plate vulcanizer at 140 °C temperature under the pressure being raised by 5 MPa per 5 min from 0 to 15 MPa; then, the uniformly melted material is irradiated by a light source array of UV LED units (NVSU233A-U365, Riya Electronics Chemistry Co., Ltd., Shanghai, China) for 2 s on an irradiation platform at normal pressure and room temperature. The XLPE materials with the crosslinking processes initiated by BP/TAIC or BPL/STAIC are finally prepared after being short-circuit hot-degassed at 80 °C for 48 h in a vacuum oven.

According to the infrared absorption peaks for characteristic molecular groups of LDPE, BPL, and STAIC as listed in Table 1 [35,36], the molecular structures of XLPE samples prepared from LDPE/BPL and LDPE/BPL/STAIC systems are characterized by Fourier Transform Infrared (FT-IR) Spectroscopy (FT/IR-6100, Jiasco Trading Co., Ltd., Shenyang, China) in a spectral range of 500–4000 cm^−1^ with a scanning resolution of 2 cm^−1^. The alternating current dielectric breakdown strength (DBS) of the circular film samples with a diameter of 50 mm and a thickness of 0.1 mm are tested by asymmetric columnar electrodes (20 mm and 40 mm in diameter for high voltage and ground electrodes, respectively) in simethicone at ambient condition. The maximum voltage is recorded before the sample being breakdown when the applied electric field is raised continuously at a constant speed of 1 kV/s, the data of which are finally analyzed with Weibull statistics.

## 3. Results and Discussion

### 3.1. Photon-Initiated Free Radicals

**Figure 2:**
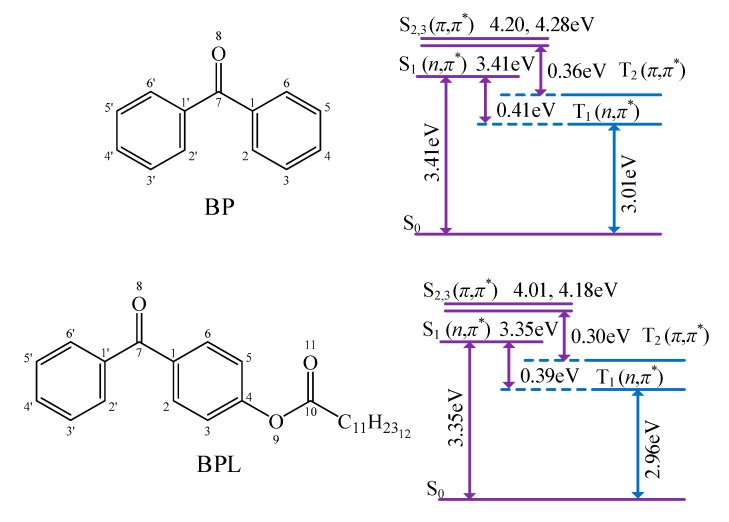
The electronic excited states and transitions of benzophenone (BP) and BPL.

The photon initiators of BP and BPL present a carbonyl double bond and a lone pair of electrons, which can be excited by UV photons from the ground state *S*_0_ to the first singlet state *S*_1_(n,π*), to the second singlet state *S*_2_(π,π*), to the first triplet state *T*_1_(n,π*), and to the second triplet state *T*_2_(π,π*) with the calculated excitation energies shown in Figure 2. The spontaneous transition time of internal conversion *S*_2_→*S*_1_ is only about 10^−12^ s, while the inter-system crossing (ISC) of *S*_1_→*T*_1_ needs 10^−10^ s, which means that inter-system crossing through *S*_2_/*S*_1_/*T*_1_ three-state intersection can occur with a high rate. After photon excitation, the free radicals on PE molecular chains can be generated by hydrogen abstraction from PE molecules to active carbonyl of photon-initiators in *T*_1_ excited state [37,38]. Since the benzene-bridged carbonyl on BP is the functional group to initiate the hydrogen abstraction reaction, the photon excitation and hydrogen abstraction processes will be identically initiated by UV irradiation on BPL, which maintains this functional group. The BPL molecule with a larger molecular weight than that of BP possesses a long alkyl chain with high compatibility to polyethylene, leading to attenuated evaporation and higher initiation efficiency in the process of generating free radicals on PE molecules. Compared with BP, the energy levels of BPL in the *S*_1_ and *T*_1_ states are closer, which implies a higher ISC frequency of *S*_1_→*T*_1_. Therefore, a higher density of *T*_1_ excited states can be produced by UV irradiation in BPL molecules than in BP, resulting in a higher reaction rate of hydrogen abstraction to achieve more free radicals on polyethylene molecules. In order to further explore the efficiency and performance mechanism of producing free radicals by photon initiators, the charge populations of BP and BPL in the ground state and excited states are analyzed by Mulliken atomic charges, as the calculated results listed in Table 2. In comparison to the ground state *S*_0_ and excited state *S*_1_, the photon initiators in excited state *T*_1_ provide double radicals on ketone carbonyl (C=O) as indicated by the significantly higher positive and lower negative Mulliken charges on C_7_ and O_8_ atoms (the labeled numbers for each atom are shown in Figure 2) respectively, while the charges populated on other carbon atoms are almost unchanged. These photon-excited variations of charge population in BPL are more appreciable than in BP, which means that a higher reaction activity of C_7_ and O_8_ in BPL for causing the partial fracture of the ketone C=O double bond will engender the higher efficiency of producing free radicals and subsequent hydrogen abstraction by the oxygen atom. Eventually, in addition to the direct crosslinking by forming chemical bonds between free radicals on PE molecules, the radical PE molecules and the hydrogenated BPL with a free radical on C_7_ will coordinately react with the auxiliary crosslinking agent such as STAIC to fulfill the indirect crosslinking of PE molecules through the partial fracture and hydrogenation of allyl double bonds on STAIC molecules.

The stationary energies and geometries of the reactants, transition states, and products of BPL photon-initiated free radical generations are optimized by minimizing the total energy based on the conjugated gradient algorithm. The fractured or formed lengths of carbon–hydrogen (C–H) and oxygen–hydrogen (O–H) bonds in hydrogen abstraction and hydrogenation processes, the broken/forming ratios of hydrogen bond length at transition states and their imaginary frequencies, and the energy barriers and Gibbs free energies of radical-generating reactions are calculated, as shown by the results listed in Table 3. Each transition state has only one corresponding imaginary frequency of the broken or bonding vibration mode, while the harmonic oscillations of the reactants and products are all real frequencies. BPL in a *T*_1_ excited state (BPL^T1^) produces the PE molecules with a secondary carbon free radical (C_2_-PE*) or tertiary carbon radical (C_3_-PE*) by the hydrogen abstraction reaction, while the hydrogenated BPL with a free radical on the carbonyl carbon atom (BPL^*H^) will cause the partial fracture and free radical of carbon double bonds on TAIC/STAIC molecules through donating hydrogen. In these hydrogen transfer reactions generating free radicals on PE and STAIC molecules, the elongation of the broken bond is smaller than that of forming a bond at the transition state, which means that the bonding pathway of O–H to BPL or C–H to an auxiliary crosslinking agent is longer than the breaking process of C–H from the PE molecule or OH from BPL^*H^, as shown in Table 3. Based on Hammond’s hypothesis, these radical-generating reactions being accompanied with hydrogen transfer, which engender C_2_-PE*, C_3_-PE*, TAIC* (as shown in Figure 1), and STAIC with free radicals (STAIC*) for PE crosslinking, are expected to be exothermic. According to the height of the reaction energy barrier (*E*_b_), which indicates the difficulty of breaking the C–H or O–H bond, it can be predicted that the reaction path producing C_2_-PE* is the main reaction channel of hydrogen abstraction due to its remarkably lower energy barrier than the C_3_-PE* path. Negative values of reaction Gibbs free energy (Δ*G* < 0) indicate that all four reactions can occur spontaneously, in which the BPL^*H^ + STAIC reaction represents the superiority in both thermodynamics and kinetics due to the lower *E*_b_ and Δ*G* than the BPL^*H^ + TAIC reaction. To this end, the BPL photo-initiation reactions of generating free radicals can be realized primarily by the BPL^T1^ abstracting hydrogen atom on the non-branched-chain of PE molecules and then partially breaking the allyl double bond by hydrogenating the auxiliary crosslinking agents (STAIC is more preferable than TAIC).

### 3.2. Crosslinking Reactions of Radical Polyethylene Molecules

The bond length of the unsaturated C–C bonds on the reactant auxiliary crosslinking agent with radicals, the bond length of the produced C–C bonds connecting the crosslinked PE molecule or the homopolymerized auxiliary crosslinking agent, and the Gibbs free energy in the crosslinking of radical PE molecules (C_2_-PE*) and the homopolymerization of radical auxiliary crosslinking agent (STAIC* or TAIC*) are calculated with the results listed in Table 4. For all the crosslinking reaction paths, the vibration frequency of each product is the real frequency. The direct crosslinking between PE molecules by forming chemical bonds with free radicals can occur with the smallest thermodynamic driving force (smallest absolute Δ*G*), while the indirect crosslinking reactions of C_2_-PE* through the monomer and homopolymer of the auxiliary crosslinking agent are more spontaneous in thermodynamics with a larger absolute Δ*G*. Due to the instability of free radicals on PE molecules and the steric effect, the homopolymerization reactions of auxiliary crosslinking agents with free radicals is more easily carried out than their crosslinking reaction with radical PE molecules, as indicated by the lower negative Δ*G* of homopolymerization reactions than that of PE auxiliary crosslinking reactions. Therefore, the PE-[AC]-PE crosslinking structure is preferred in the crosslinking reactions of C_2_-PE* with auxiliary crosslinking agents. Besides, compared with TAIC*, the STAIC* will connect the C_2_-PE* with a higher reaction rate also due to the steric effect, as demonstrated by the lower negative Δ*G* shown in Table 4.

### 3.3. Energetic Electronic Properties of Molecules in Crosslinking Reactions

**Figure 3:**
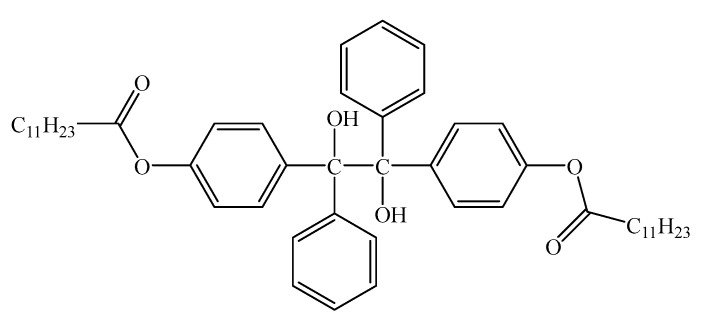
The molecular schematics of by-product PBBL produced by BPL in a UV-initiated crosslinking reaction of polyethylene.

In order to estimate the electrical properties of the UV-initiated XLPE, the ionization potential (IP) and electronic affinity (EA) of the reactants and by-product molecules in a photon-initiated crosslinking reaction, and the electron energy bandgap *E*_g_ = *E*_LUMO_ − *E*_HOMO_ (*E*_LUMO_ and *E*_HOMO_ denote the energy levels of the lowest unoccupied molecular orbital and the highest occupied molecular orbital respectively) are analyzed by the first-principles electronic structure calculations, according to the results listed in Table 5. The main by-product produced by BPL in a UV-initiated polyethylene crosslinking reaction is named PBBL with a molecular structure as schematically shown in Figure 3. The EA values of reaction by-products containing carbonyl, hydroxyl, or ester groups is significantly higher than that of the PE molecule, which thereby can capture hot electrons and increase dielectric breakdown strength of XLPE. The IP values of STAIC, BPL, and PBBL are less than that of TAIC, BP, and PBB (by-product from BP, as illustrated in Figure 1), respectively, which can more efficiently prevent the degradation of PE matrix. The STAIC, BPL, PBBL macromolecules have higher EA than that of acetophenone (AP, a typical voltage stabilizer), and thus can capture high-energy electrons in efficiency and convert the hot-electronic energy into atomic vibration through the ketone enol isomerization reaction of the ester carbonyl group (electron–phonon coupling), which acts as a voltage stabilizer [39]. Despite of possessing a notably higher EA than AP, the BP with a minimal residual content in XLPE materials cannot actually contribute to voltage stabilization due its high volatility and low compatibility with PE. Finally, to verify the validity of using the M11-L functional and the accuracy of the calculated results, the calculations of *E*_g_, IP, and EA are also performed by employing hybrid functional B3LYP with the same schemes of core treatment, basis set, SCF tolerance, and geometry optimization control as by employing the M11-L functional, with the calculated results being listed in Table 5 for comparison. The B3LYP results do not show explicit discrepancy (less than 6% in difference) compared with the M11-L results, demonstrating that DFT calculations adopting the improved local DFT functional M11-L as a meta-GGA form can achieve adequately accurate electronic structure results. 

### 3.4. Infrared Spectrum Characterization

**Figure 4:**
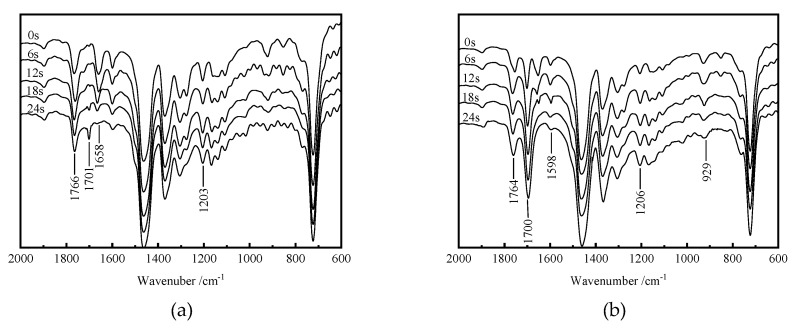
Infrared spectra of photo-initiated crosslinking reaction systems: (**a**) LDPE/BPL; (**b**) LDPE/BPL/STAIC.

In order to investigate the variation of characteristic groups with the prolongation of UV irradiation in the crosslinking reaction using a macromolecular photon-initiation system (BPL and STAIC), the infrared spectra of LDPE/BPL and LDPE/BPL/STAIC systems for UV-initiated crosslinking reactions are tested at various irradiation time, and the results are shown in Figure 4. The characteristic absorption peak of hydroxyl on the BPL by-product, which is produced in the photon-initiated reactions of generating free radicals, appears at 1701 cm^−1^ with the peak intensity increasing gradually as the UV irradiation time is raised. In addition, with the prolongation of UV irradiation, the characteristic peak of the ketone carbonyl group (1658 cm^−1^) in the LDPE/BPL system gradually decreases and finally disappears, while the characteristic peaks of the ester carbonyl group (1766 cm^−1^) and ester group (1203 cm^−1^) show no obvious change in absorption intensity. Thus, it is concluded that the macro-photon-initiator BPL with a long alkyl chain maintains the photon-initiation characteristics of BP due to the benzene hydroxyl functional group, which is gradually consumed to form a reaction by-product, while the alkyl long-chain of BPL has not participated in the photon-crosslinking reaction.

For the photon-initiated PE crosslinking system of LDPE/BPL/STAIC, a peak fading away with UV irradiation has also been observed at 1598 cm^−1^ and 929 cm^−1^ which derives from the allylic double bond of STAIC (as listed in Table 1). In contrast, the 1700 cm^−1^ characteristic peak (identical to that of the phenylcarbamoyl group) from the phenylcarbamoyl vibration of the BPL-introduced by-product has been substantially intensified by raising the irradiation time, while the ester-carbonyl and ester groups of STAIC remain unchanged in density as characterized by their constant vibration peaks at 1764 cm^−1^ and 1206 cm^−1^, respectively. Therefore, despite possessing multiple functional groups, STAIC contributes to the crosslinking reaction only by allyl double bonds, which have been gradually consumed in the auxiliary crosslinking reactions.

### 3.5. Dielectric Breakdown Strength

**Figure 5:**
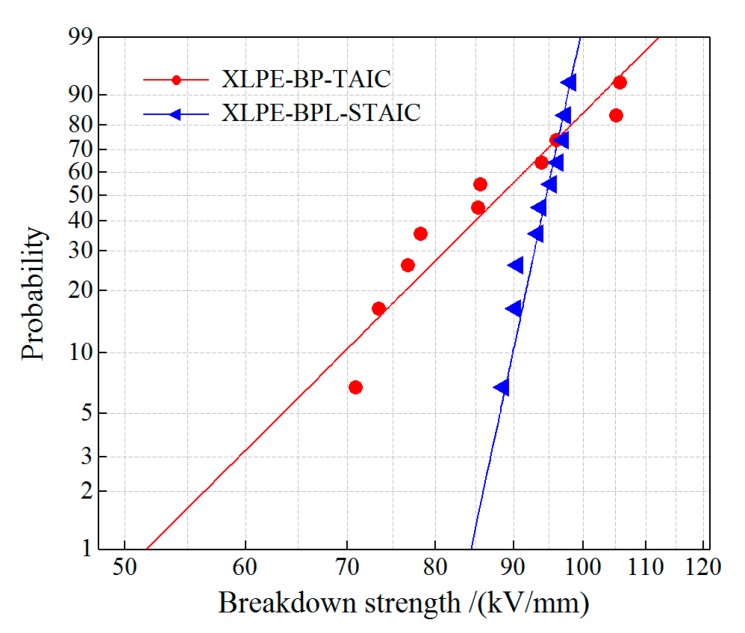
Dielectric breakdown strength statistics fitted with two-parameter Weibull distribution for XLPE samples prepared by different photon-initiation systems.

Dielectric breakdown experiments have been performed for the UV-initiated XLPE materials using the macromolecular and micromolecular photon-initiation systems, which are represented by XLPE-BP-TAIC and XLPE-BPL-STAIC, respectively. The tested DBS results are analyzed by Weibull statistics fitted with two-parameter distribution, as shown in Figure 5. The shape parameters and characteristic breakdown field of the two kinds of XLPE film samples are listed in Table 6. The characteristic breakdown field of the XLPE-BPL-STAIC material is significantly higher than that of the XLPE-BP-TAIC material. In particular, the shape parameter of the XLPE-BPL-STAIC material has been greatly improved in comparison to XLPE-BP-TAIC, which means that the numerical distribution of the electrical breakdown field is more concentrated, demonstrating that the higher voltage stability has been acquired by the XLPE-BPL-STAIC material. The experimental DBS results are consistent with the theoretical prediction that the ester carbonyl group presented by employing a macromolecular photon-initiation system can act as a voltage stabilizer.

## 4. Conclusions

In order to elucidate the underlying physical and chemical mechanisms of UV-initiated polyethylene crosslinking reactions, the excited states of the photon initiator, the transition states, the energies of radical producing and connecting reactions, and the electronic structure of crosslinking by-products are calculated by the all-electron numerical orbit first-principles method for both micromolecular and macromolecular photon-initiation systems. Accordingly, XLPE materials are prepared employing the UV-initiation crosslinking technique and tested by infrared spectroscopy and dielectric breakdown experiments to verify the insulation performance improvement caused by using a macromolecular photon-initiation system. The macromolecular photon initiator BPL with a polyethylene-compatible alkyl chain can effectively initiate the crosslinking reaction of polyethylene molecules under UV irradiation through the monomer or homopolymer of the macromolecular auxiliary crosslinking agent STAIC, as to form a crosslinking network with the STAIC as the crosslinking nodes. The results of infrared spectroscopy indicate that the benzene-bridged carbonyl group of BPL and the allyl double bond of STAIC are gradually consumed with the prolongation of UV irradiation to form by-products and crosslinking nodes respectively, which qualitatively confirm the theoretical calculation results. The residual reactants and by-products of BPL-initiated STAIC auxiliary crosslinking reaction can capture high-energy electrons in higher efficiency than the representative voltage stabilizer, and thus can act as a voltage stabilizer to convert the energy from hot electrons into atomic vibration through the ketone–enol isomerization reaction of ester carbonyl group. The dielectric breakdown experiments imply that the insulation performance of XLPE is ameliorated by using BPL/STAIC in comparison to BP/TAIC, which is consistent with the first-principles electronic structure calculations. The present study renders a prospective routine for developing UV-crosslinking technology, which can be applied to high-efficiency industrial production, and it demonstrates a technical strategy to improve the insulation performance of XLPE materials by exploiting high-efficiency photon-initiator and functional crosslinking agent.

## Figures and Tables

**Table 1:** Infrared absorption peaks of characteristic groups on the reactant molecules in a photon-initiated polyethylene crosslinking reaction. BPL: 4-hydroxybenzophenone laurate, STAIC: dioleyl-2,2-,4-,4-tetraallyl isocyanurate, LDPE: low density polyethylene.

Reactant Molecules	Characteristic Groups		Absorption Peaks/cm^−1^
BPL	ketone carbonyl –C=O		1658
	ester carbonyl O–C=O		1766
	ester C–O–C		1203
STAIC	allyl double bond =CH_2_/C=C		929/1598
	phenylcarbamoyl N–C=O		1700
	ester carbonyl O–C=O		1764
	ester –C–O–C–		1206
LDPE	alkyl –CH_2_-	stretching	2898
		bending	1460
	alkyl –C_4_H_9_		720

**Table 2:** Mulliken atomic charges of BP and BPL.

Photon Initiator	States	Mulliken Charges/e										
		C_1_/C_1′_	C_2_/C_2′_	C_3_/C_3′_	C_4_/C_4′_	C_5_/C_5′_	C_6_/C_6′_	C_7_	O_8_	O_9_	C_10_	O_11_
BP	*S*_0_	0.04	−0.14	−0.09	−0.08	−0.08	−0.09	0.31	−0.39			
	*S*_1_	0.05	−0.15	−0.09	−0.08	−0.08	−0.09	0.33	−0.41			
	*T*_1_	0.06	−0.15	−0.08	−0.09	−0.07	−0.09	0.39	−0.50			
BPL	*S*_0_	0.03/0.04	−0.14/−0.14	−0.13/−0.09	0.34/−0.08	−0.14/−0.08	−0.09/−0.09	0.33	−0.40	−0.41	0.51	−0.37
	*S*_1_	0.04/0.06	−0.12/−0.12	−0.13/−0.08	0.32/−0.11	−0.12/−0.08	−0.10/−0.12	0.34	−0.41	−0.39	0.51	−0.38
	*T*_1_	0.05/0.06	−0.13/−0.13	−0.13/−0.08	0.31/−0.10	−0.12/−0.09	−0.11/−0.13	0.42	−0.51	−0.40	0.50	−0.38

**Table 3:** Reactant C–H (or O–H) bond length *L*_R_ (Å) and product O–H (or C–H) bond length *L*_P_ (Å), hydrogen bond broken/forming length ratio *R*_b/f_ and imaginary frequency *f*_i_ (cm^−1^) of the transition state, energy barrier *E*_b_ (eV), Gibbs free energy Δ*G*^0^_298_ (kcal/mol) in photon initiations of free radicals.

Schematic Molecular Reactions	*L*_R_	*R*_b/f_	*L*_P_	*f*_i_	*E*_b_	Δ*G*^0^_298_
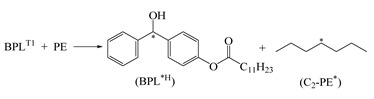	1.098	0.881	0.958	1247	0.14	−11.833
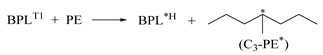	1.104	0.870	0.958	1235	0.25	−10.475
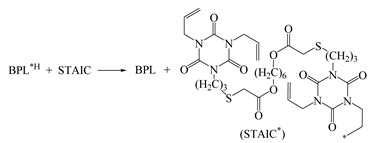	0.958	0.847	1.102	1306	0.37	−7.649
BPL^*H^ + TAIC → BPL + TAIC*	0.958	0.915	1.092	1327	0.46	−5.182

**Table 4:** The bond length *L*_R_ of the reactant C–C bonds on the radical auxiliary crosslinking agent, the bond length *L*_P_ of the produced C–C bonds connecting the crosslinked PE molecule or homopolymerized auxiliary crosslinking agent, Gibbs free energy Δ*G*^0^_298_ in crosslinking and homopolymerization reactions.

Schematic Molecular Reactions	*L*_R_/Å	*L*_P_/Å	Δ*G*^0^_298_/(kcal/mol)
2 C_2_-PE* → PE-PE		1.524	−85.655
C_2_-PE* + TAIC* → PE-TAIC	1.481	1.511	−101.096
2 TAIC* → -[TAIC]_2_-	1.481	1.497	−122.999
C_2_-PE* + STAIC* → PE-STAIC	1.476	1.517	−107.480
2 STAIC* → -[STAIC]_2_-	1.476	1.504	−131.105

**Table 5:** Energy band-gap, ionization potential, and electron affinity (all in eV unit) of photon initiators, auxiliary crosslinking agents, and by-products for UV-initiated polyethylene crosslinking reactions.

Molecules	M11-L					B3LYP				
	*E*_g_	IP(a)	IP(v)	EA(a)	EA(v)	*E*_g_	IP(a)	IP(v)	EA(a)	EA(v)
BP	3.795	8.695	8.766	0.790	0.546	3.808	8.912	8.661	0.819	0.560
BPL	3.783	8.561	8.594	0.816	0.675	3.964	8.668	8.886	0.795	0.642
TAIC	5.890	8.776	8.828	−0.461	−0.519	6.206	8.970	8.632	−0.438	−0.545
STAIC	4.366	7.767	7.823	0.458	0.372	4.635	8.216	8.165	0.426	0.358
PBB	4.390	7.976	8.065	−0.073	−0.228	4.447	8.349	8.398	−0.075	−0.216
PBBL	4.291	7.855	7.977	1.104	1.022	4.583	8.359	7.869	1.074	0.969
PE	8.232	7.644	7.701	−0.874	−0.913	8.229	7.476	7.992	−0.919	0.873
AP	3.223	8.757	8.873	0.214	0.025	3.415	8.685	9.277	0.229	0.024

**Table 6:** The characteristic 63.2% DBS and shape parameter of Weibull distribution fitted in 95% confidence interval for XLPE samples prepared with different photon initiators and auxiliary crosslinkers.

Experimental Samples	Shape Parameter	Characteristic DBS/(kV/mm)
XLPE-BP-TAIC	7.91	87.6
XLPE-BPL-STAIC	37.14	95.4
